# Time delay of the qCON monitor and its performance during state transitions

**DOI:** 10.1007/s10877-020-00480-4

**Published:** 2020-02-10

**Authors:** Robert Zanner, Gerhard Schneider, Adrian Meyer, Eberhard Kochs, Matthias Kreuzer

**Affiliations:** 1 Department of Anesthesiology and Intensive Care, Technical University of Munich School of Medicine, Ismaninger Str. 22, 81675 Munich, Germany; 2 Department of Anesthesiology, HELIOS Clinic Wuppertal, Witten/Herdecke University, Heusnerstr.40, 42283 Wuppertal, Germany

**Keywords:** Anesthesia, general, Electroencephalography, Monitoring, Humans

## Abstract

We investigated the performance of the qCON index regarding its time delay for sudden changes in the anesthetic level as well as to separate responsiveness from unresponsiveness during loss and return of responsiveness (LO*R* and RO*R*). For evaluation of the time delay, we replayed relevant EEG episodes to the qCON to simulate sudden changes between the states (i) *awake/sedation*, (ii) *adequate anesthesia*, or (iii) *suppression.* We also replayed EEG from 40 patients during LO*R* and RO*R* to evaluate the qCON’s ability to separate responsiveness from unresponsiveness. The time delays depended on the type of transition. The delays for the important transition between *awake/sedation* and *adequate anesthesia* were 21(5) s from *awake/sedation* to *adequate anesthesia* and 26(5) s in the other direction. The performance of the qCON to separate responsiveness from unresponsiveness depended on signal quality, the investigation window, i.e. ± 30 s or ± 60 s around LO*R*/RO*R*, and the specific transition being tested. AUC was 0.63–0.90 for LO*R* and 0.61–0.79 for RO*R*. Time delay and performance during state transitions of the qCON were similar to other monitoring systems such as bispectral index. The better performance of qCON during LO*R* than RO*R* probably reflects the sudden change in EEG activity during LO*R* and the more heterogeneous EEG during RO*R*.

## Introduction

Monitoring the hypnotic component of anesthesia based on frontal electroencephalographic (EEG) recordings during surgical interventions has gained increasing popularity over the last twenty years.

### Monitoring the hypnotic component of anesthesia

Monitoring devices calculate an index based on processed EEG parameters, mainly derived from the frequency domain. The most common monitor is the bispectral index (BIS, Medtronic, Dublin, Ireland) that uses information from the power spectrum as well as from higher order spectra [1]. Other available devices are the Entropy Module (GE Healthcare, Little Chalfont, UK) [2], the Narcotrend (NCT, MonitorTechnik, Bad Bramstedt, Germany) [3], the Index of Consciousness (IoC; Morpheus Medical, Barcelona, Spain) [4], or the patient state index (PSI, SEDLine, Masimo, Irvine, CA) [5]. These devices also evaluate changes in the EEG frequency composition that are induced by many of the common anesthetics, namely a shift from a low amplitude, high frequency signal in a responsive patient to a slow rhythm with high amplitudes during anesthesia [6]. The benefit of using these indices to monitor anesthesia has been subject to controversial discussion [7, 8]. One point of criticism is a possible inability of the indices to detect episodes of intraoperative awareness, especially in patients with neuromuscular block [9, 10]. Another issue is the time delay of index calculation that may prevent the anesthesiologist from timely detecting sudden changes in the anesthetic level [11–13]. These issues may hinder the indices to reliably separate conscious or responsive states from unconscious or unresponsive states on-line at the state transitions [14–16]. Specific information regarding the performance of a relatively new index, the qCON (Quantium Medical, Mataro, Spain) [17], that is now integrated in the CONOX monitor (Fresenius Kabi AG, Bad Homburg, Germany) [18], is not available to a great extent. Here we present the results regarding the time delay of the qCON as well as its ability to distinguish (goal-directed) responsiveness from unresponsiveness [19, 20] state transitions.

### The qCON

The qCON processes frontal EEG information and reflects the estimated anesthetic level as a dimensionless number between 99 (fully awake) and 0 (isoelectric EEG) and the detailed algorithm is described in the article by Jensen et al. [17] In short, the index is based on four spectral parameters that are calculated from the signal energy of different EEG frequency bands. Prior to the calculation of these parameters the recorded EEG is checked for artefacts using an artefact rejection routine. An Adaptive Neuro Fuzzy Inference System (*ANFIS*) forms the core of the algorithm that is used to evaluate the anesthetic level. In short, the single parameters represent the logarithmic energy ratio between the classical EEG theta, alpha, beta and gamma bands and the total energy in the signal frequency range from 1 to 44 Hz. The ANFIS combines these parameters and a burst suppression parameter using set rules and generates an index that corresponds with the anesthetic level. qCON indices ≥ 80 correspond to the awake state or light sedation and the index range from 60 to 40 corresponds to the anesthetic level suitable for *adequate anesthesia*. Lower indices are associated with deep anesthesia and burst suppression. In order to detect burst suppression, the qCON calculates a burst suppression ratio (*BSR*) that is defined as the fraction of suppressed EEG activity within 30 s and ranges between 0 and 100%.

In order to evaluate the performance of the qCON, we used pre-recorded EEG signals derived during loss and return of responsiveness (LO*R*/RO*R*) state transitions as well as steady state recordings that displayed stable qCON indices over a defined time span.

## Methods

We performed all of our analyses using previously recorded EEG data which were derived from patients participating in two different studies who had consented in written form to the protocol, which was approved by the ethics committee of the Technische Universität München, Munich, Germany.

In order to use the most suitable data set for transition and time delay analysis, we used two different data sets.

### Data for time delay estimation

For the task of evaluating the time delay, we used EEG episodes from the study by Horn et al. [21] that led to a stable index behavior over 5 min. This study was designed to keep the anesthetic at constant concentrations for 15 min, allowing for the extraction of EEG episodes with a stable index.

### Data for evaluation of qCON at state transitions

For the evaluation of the qCON performance at the state transitions, we replayed data from a previously published study by Schneider et al. that was designed to evaluate the bispectral index at the state transitions during anesthesia induction and awareness as well as during a short episode of simulated awareness [15]. In short, the EEG was recorded with a sampling rate of 1 kHz and a band pass from 0.5 to 400 Hz from frontal positions using a BIS A-1000 monitor.

EEG data from both studies has been stored in an institutional, custom made database [22]. Selected EEG episodes were played back to the qCON using a custom made device [23].

#### Evaluation of time delay

For evaluation of the time delay of index calculation of the qCON following sudden, simulated, changes between different anesthetic levels, we first searched for EEG episodes of 5 min that led to stable indices reflecting the states *awake/sedation* and *adequate anesthesia*. In accordance with the qCON guidelines (https://quantiummedical.com/products/qcon2000/), qCON values between 40 and 60 reflect *adequate anesthesia* and qCON values above 80 indicate *awake/sedation*. For our analyses, we used five of these segments from five different patients for each level, i.e., *awake/sedation* and *adequate anesthesia*.

For simulation of the very deep anesthesia level *suppression,* reflected by an isoelectric EEG suppression signal, we used a zero line. The five 5 min episodes leading to stable *awake/sedation* indices and the five 5 min episodes leading to stable *adequate anesthesia* indices were concatenated in all possible 25 combinations in order to evaluate sudden state transitions from (i) *suppression* to *awake/sedation* and back, (ii) *suppression* to *adequate anesthesia* and back, as well as (iii) *adequate anesthesia* to *awake/sedation* and back. We measured the qCON time delay for each transition as the time span of the qCON from the sudden change of state to the display of an index value representing the new state.

In contrast to our experiments with other monitors of the hypnotic component of anesthesia [13, 19] the recorded EEG sequences extracted from our database did not all produce nearly stable qCON index values. This different behavior may be due to the different approaches the devices calculate the index. We were therefore not able to establish a reference target index value as described previously [11, 13]. Hence, we used the qCON ranges for *awake/sedation* and *adequate anesthesia* as given by the manufacturer.

The target index ranges were qCON of 80 or above for *awake/sedation*, qCON between 40 and 60 for *adequate anesthesia*, and a qCON below 3 for *suppression*. The concatenated EEG sequences *suppression* to *awake/sedation* to *suppression* (*n* = 5) and *suppression* to *adequate anesthesia* to *awake/sedation* to *adequate anesthesia* to *suppression* (*n* = 25, five times five possible combinations between *adequate anesthesia* and *awake/sedation* EEG sequences) were replayed three times and the complete results were used to determine the time delay.

#### Evaluation of the qCON at LOR and ROR

To evaluate the performance of the qCON to distinguish between responsiveness and unresponsiveness at the state transitions LO*R* and RO*R* we used pre-recorded EEG from a different data set recorded with the intention to evaluate processed EEG during LO*R* and RO*R*. State transitions were assessed by the anesthesiologist as loss (LO*R*) or return (RO*R*) of a repeated response to verbal command as previously described. The repeated response to a command correlates to a state of goal-directed responsiveness [20]. The generation of these EEG data and the clinical protocol are described in detail elsewhere [15]. Briefly, 40 unpremedicated patients scheduled for elective surgery were randomly assigned to receive either sevoflurane/remifentanil or propofol/remifentanil anesthesia. During smooth induction with either sevoflurane or propofol, patients were asked every 30 s to squeeze the hand of the investigator. Failure to respond to command was defined LO*R*1. Then, Tunstall’s isolated forearm technique [24] was employed to further assess responses after neuromuscular blockade with succinylcholine. After tracheal intubation, administration of sevoflurane or propofol was discontinued until response to command returned (RO*R*1). Subsequently, delivery of anesthetic drugs was resumed. Cessation of response indicated LO*R*2. At the end of surgery, anesthetic drugs were discontinued and the first response of the patient to verbal command marked RO*R*2. Because this intermediate state the patient regained responsiveness (RO*R*1/LO*R*2) was a very dynamic process of short duration, we refrained from including these transitions in our analysis and only used LO*R*1 and RO*R*2. In order to consider the signal quality of the EEG we used the information provided by the qCON monitor as signal quality index (SQI). The SQI ranges from 0 to 100% and provides information regarding the reliability of the qCON index. In previous studies SQI < 50 were excluded from analyses because of low signal quality [17, 25]. In accordance, we evaluated the performance of the qCON for the (i) entire data set, (ii) only cases with SQI > 50, and (iii) SQI > 75.

In order to evaluate possible differences in the qCON performance to track the transition during this highly dynamic phase we chose to extract the qCON indices at two different time points (30 and 60 s) before and after the transition. We then compared the indices extracted 30 s before the transition (− 30 s) with the indices 30 s after (+ 30 s) the transition and we reran the analyses for the ± 60 s setting.

### Statistical analysis

We chose to present the results from the time delay analysis in a descriptive form. For the evaluation of the ability of qCON to distinguish responsiveness from unresponsiveness during LO*R* and RO*R* we calculated the area under the receiver operating characteristics curve (AUC) with 10 k-fold bootstrapped 95% confidence intervals using the MES toolbox [26]. For dichotomous data as in our case the AUC is identical to the prediction probability (PK) [27]. AUC ranges between 0.5 and 1 or 0 and 1 if the direction is considered. For our analyses, AUC = 1 means that every qCON index can be assigned to either responsiveness or unresponsiveness with 100% certainty. An AUC = 0.5 means that the assignment of a qCON to one of the states is by chance. As a role of thumb, AUC ≥ 0.7 seems to present an effect (performance) of relevance [28]. The performance in the range of AUC ≥ 0.7 to AUC = 1 may be categorized as AUC = [0.90–1]: *excellent*, AUC = [0.80–0.90]: *good*, AUC = [0.70–0.80]: *fair *[29]. We used MATLAB R2017b to create the plots and Inkscape 0.48 to edit the figures.

## Results

### Time delay of the qCON

**Fig. 1:**
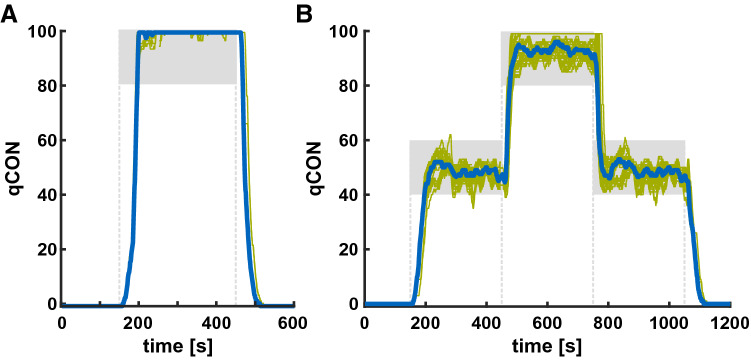
Time delay of state transitions. The qCON showed different time delays for the different state transitions. **a** For the transition from *suppression* to a*wake/sedation* and back, the delay was 45(2) s and 52(4) s. **b** For the transition from *suppression* to *adequate anesthesia* and back, the delay was 46(4) s and 52(4) s. For the transition from *adequate anesthesia* to a*wake/sedation* and back, the delay was 26(5) s and 21(5) s. The blue lines indicate the median and the green lines indicate the single replays. The grey squares indicate the target region. Delays in the legend are presented as mean and standard deviation

The mean time delay of the qCON for the single transitions ranged from 21 to 52 s. The fastest transitions were from *awake/sedation* to *adequate anesthesia* and back. The slowest transitions were from both *awake/sedation* and *adequate anesthesia* back to *suppression*. Table 1 presents the detailed delays for the single transitions. Figure 1 displays the course of qCON index values during the experiments.

**Table 1:** Time delays of index calculation at the different transitions

Transition	Target qCON range	Time delay (s) (mean (SD))	Time delay (s) [median (range)]
Suppression → adequate anesthesia	qCON ≥ 40	46 (4)	44 (41–67)
Suppression → awake/sedation	qCON ≥ 80	45 (2)	45 (41–47)
Adequate anesthesia → awake/sedation	qCON ≥ 80	26 (5)	25 (16–38)
Awake/sedation → adequate anesthesia	qCON ≤ 60	21 (5)	20 (11–36)
Adequate anesthesia → suppression	qCON < 3	52 (4)	51 (48–63)
Awake/sedation → suppression	qCON < 3	52 (4)	50 (49–62)

The fastest adaption to the changed state occurs at transitions between *awake/sedation* and *adequate anesthesia* in the 20 s to 30 s range. The slowest transitions are towards *suppression* with delays above 50 s

### Performance of qCON between LOR and ROR

Out of the 40 patients, we were able to include 38 patients for evaluating the performance at LO*R* and 34 patients for evaluating the performance at RO*R*. This number decreased with increasing SQI threshold. For LO*R* we had 28 patients with SQI > 50 and 23 patients with SQI > 75. We could include 33 patients for both SQI thresholds for RO*R*.

In general, we found better performance of the qCON during LO*R* than at RO*R*. The performance depended on the quality of the EEG signal as evaluated by SQI as well as for the time the qCON was used for analysis before and after LO*R* or RO*R* (± 30 s, ± 60 s). We found highest AUC for the data with SQI > 75 and the ± 60 s setting. For LO*R*, this AUC was 0.90 [95% CI 0.77–0.99] and it was 0.79 [0.67–0.90] for RO*R*. Table 2 presents the AUC values for all settings used.

**Table 2:** AUC of the LO*R*/RO*R* transitions for different temporal and SQI settings

	LO*R* (± 30 s)	LO*R* (± 60 s)	RO*R* (± 30 s)	RO*R* (± 60 s)
All data	0.63 [0.50–0.75]	0.74 [0.62–0.85]	0.61 [0.47–0.74]	0.76 [0.63–0.87]
SQI > 50	0.69 [0.52–0.84]	0.85 [0.72–0.95]	0.61 [0.47–0.74]	0.76 [0.63–0.87]
SQI > 75	0.76 [0.58–0.92]	0.90 [0.77–0.99]	0.62 [0.47–0.77]	0.79 [0.67–0.90]

The performance of qCON to separate responsiveness from unresponsiveness as indicated by AUC and 95% confidence intervals was dependent on the signal quality (SQI) and the time qCON was extracted (± 30 s, ± 60 s). In general, AUC was higher for LO*R* than for RO*R*

**Fig. 2:**
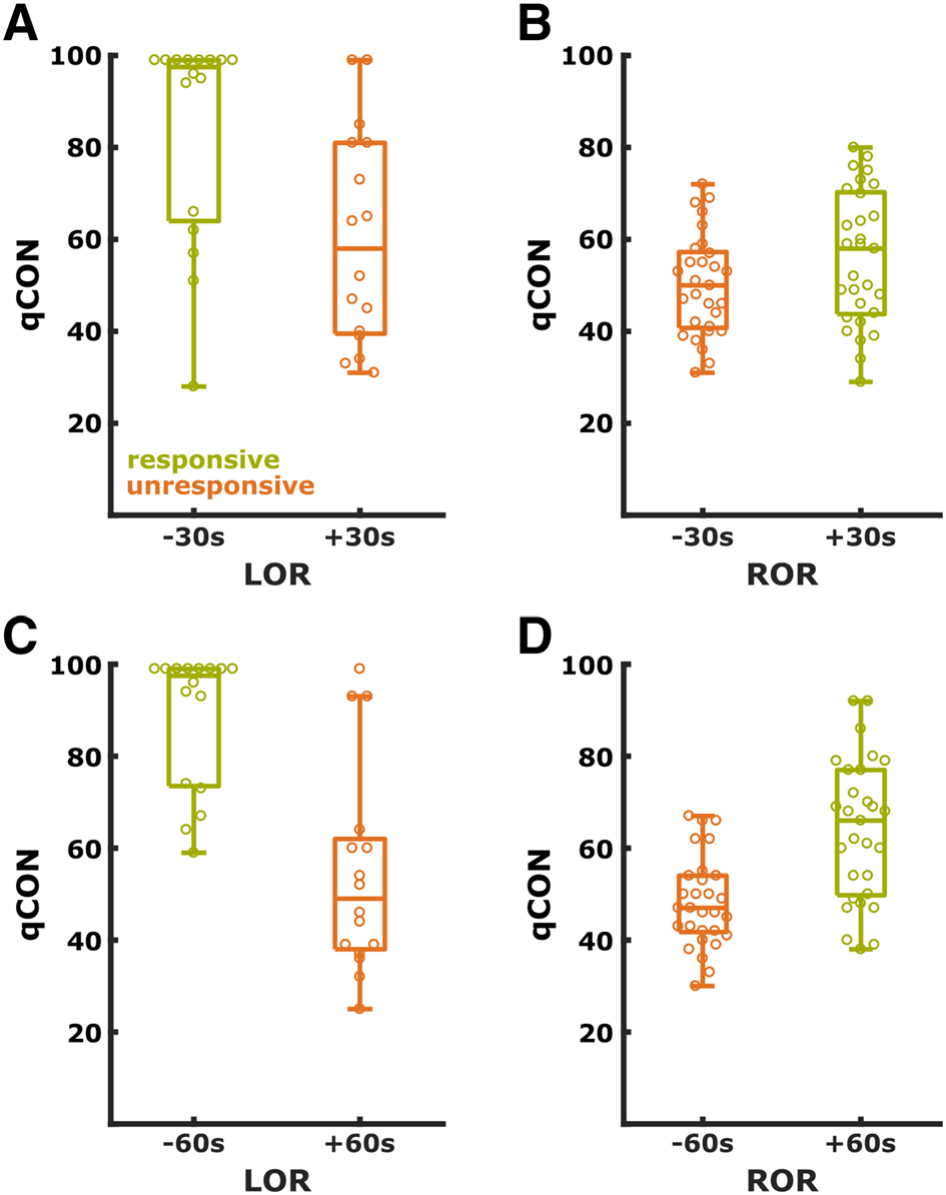
Performance of qCON at the state transitions. Performance (AUC) for the qCON at the LO*R* (left, **a**, **c**) and RO*R* (right, **b**, **d**) transition for different time settings, i.e., the comparison from qCON obtained 30 s before and after LO*R*/RO*R* (top, **a**, **b**) and 60 s before and after LO*R*/RO*R* (bottom). The single dots represent individual cases. Only cases with a signal quality > 75 were included. *LOR* Loss of responsiveness, *ROR* Return of responsiveness;

In order to depict the differences, Fig. 2 displays the performance of qCON at LO*R* and RO*R* for the ± 30 s and ± 60 s setting when we only used the EEG recordings with a SQI > 75 during LO*R* or RO*R*.

**Fig. 3:**
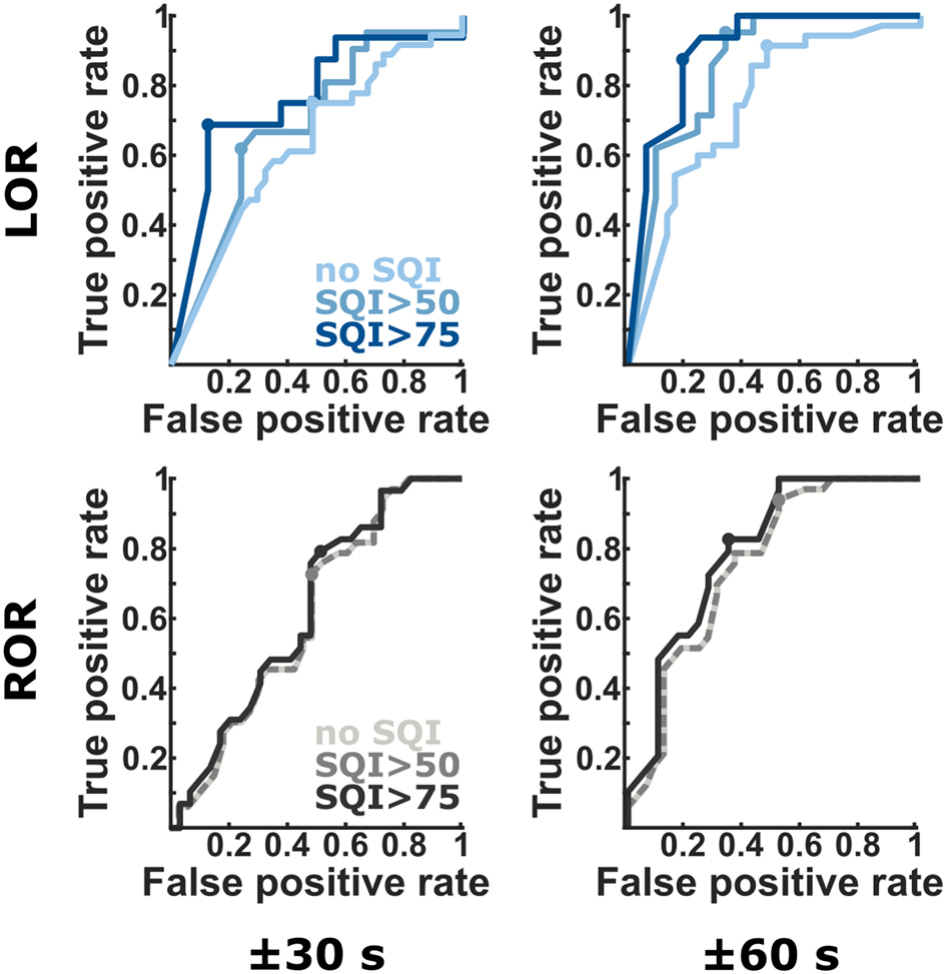
AUC curves. The performance at LO*R* was strongly dependent on signal quality that was not the case at RO*R*, mainly due to better SQI at RO*R*. Further the performance was better for LO*R* as it was for RO*R* and the longer the time between even (LO*R*/RO*R*) and the extracted qCON values, the better was the performance. The dots indicate the optimal operating point. *LOR* Loss of responsiveness, *ROR* Return of responsiveness, *SQI* signal quality index

Figure 3 displays the AUC curves for the different SQI and temporal settings (± 30 s, ± 60 s) for LO*R* and RO*R* and highlights the different. SQI-dependent performances. *LOR* Loss of responsiveness, *ROR* Return of responsiveness, *SQI* signal quality index.

## Discussion

The qCON monitor is a relatively new device with only a limited number of published investigational reports. A previous study investigated the performance of qCON and BIS in detecting the loss of consciousness defined as loss of eyelash reflex. The authors compared mean qCON values derived over 1 min immediately before starting the infusion pumps with mean qCON values derived over 1 min immediately after the loss of eyelash reflex. They determined a PK of 0.92 for qCON and 0.94 for BIS [17]. In a conference abstract, Valencia et al. described that qCON performance during loss of eyelash reflex is similar to BIS [30]. In addition, a similar performance of the qCON compared to BIS has been reported for sedation during bronchoscopic interventions [31].

### Time delay of the qCON

The qCON shows a time delay between around 20 and 50 s depending on the transition between the different levels. It has the longest delay for the transitions from either *adequate anesthesia* or *awake/sedation* to *suppression* and the shortest delay for the transitions from *adequate anesthesia* to *awake/sedation* as well as from *awake/sedation* to *adequate anesthesia*. These transitions between *adequate anesthesia* and *awake/sedation* are probably of the highest clinical interest, because they reflect the transition between consciousness/responsiveness and unconsciousness/responsiveness and vice versa. If the monitored patient for instance shows an unwanted waking (EEG) response, the anesthesiologist should react to it as soon as possible because longer episodes of wakefulness are associated with a higher risk for intraoperative recall [32]. The longer time delays into and out of suppression may be due to the detection algorithm for suppression that requires 30 s of EEG information for calculation of total suppression [17].

In comparison to the previously published data for IoC [11] the qCON adapts significantly faster to a change of the anesthetic level. It also has a significantly shorter delay than reported delays for the State Entropy (SE) of the Entropy Module in the transitions from *suppression* to *adequate anesthesia* or *awake/sedation*, from *awake/sedation* to *adequate anesthesia*, and from *awake/sedation* to *suppression* [11]*.* Since we were only able to evaluate the time delay of the qCON to reach the relevant index interval, a direct comparison to the previously presented time delays of BIS and NCT is not possible. For these monitors the time delay to reach a stable index value has been evaluated previously [12, 13]. But the delays still seem to be in a comparable range. The BIS requires 25 s (NCT: 49 s) to adequately react on the transition from *adequate anesthesia* to *awake/sedation* and 25 s (NCT: 24 s) for the backward transition. Taken together, the time delay of the qCON to sudden changes of the anesthetic levels is rather fast compared to other monitors but still considerable. In addition, the minimum/maximum delays we found for each transition could be different by around 20 s. One possibility for these differences could be based in the way the ANFIS is generating the indices. Hence, this time delay of the qCON may impair timely detection of intraoperative awareness and raise trouble if it is used for pharmacodynamic modelling, especially since the time delays are different for different transitions.

### Performance of qCON during state transitions

We observed better performance of qCON to separate responsiveness from unresponsiveness dependent on the EEG signal quality as well as on the temporal distance of the extracted qCON values to the LO*R*/RO*R* transition. When only considering EEG with very high signal quality (SQI > 75) the performance for distinguishing responsiveness from unresponsiveness during LO*R* was *fair,* according to the AUC classification as described in the *Statistical Analysis* section*,* for the qCON when extracted 30 s before and after LOC and it was *excellent* for the ± 60 s setting. For RO*R*, the performance was *fail,* for the ± 30 s and *fair* for the ± 60 s setting. In order to compare the performance of the qCON with other monitors, we have to consider the whole data set, because in other studies evaluating different monitoring systems, the performance was not evaluated for different SQI thresholds. Under these conditions, the performance of qCON at LO*R* and RO*R failed* at the ± 30 s setting and was *fair* in the ± 60 s setting. For similar scenarios, the BIS, the patient state index, as well as the NCT showed rather poor performance as well [16, 33, 34]. The State Entropy seemed to perform a little better [35] in certain but not all studies investigating anesthetic-induced state transitions [34]. In general, higher AUC values indicating a better performance to separate between consciousness and unconsciousness were reported [34, 36, 37]. But these analyses were performed during less challenging conditions. These studies compared index values when the patient was fully awake versus the index at loss of consciousness [34], or during stable levels of ICU sedation [37]. Another study tracked modeled propofol effect site concentrations [36]. In contrast to the AUC derived from these studies, our analyses focused on the dynamic transition in and out of general anesthesia. Hence, our lower AUC values most probably are a consequence of the different settings.

In general, this highly dynamic episode during state transitions in combination with the reported delay of index calculation may cause these rather poor performances. The fact that the EEG during loss of consciousness shows a sudden change from fast to slow oscillatory activity [38], whereas during return of consciousness the EEG patterns of anesthesia emergence can be quite different [39, 40] seem to explain the better performance of the qCON during LO*R*.

## Limitations

We used pre-recorded EEG for our analysis of time delay and performance instead of recording the qCON directly from the patient. But since our method has been established [14, 16, 35] and other groups have presented similar technologies as well [41] we are confident that our analyses can add valuable information to the field of EEG-based depth of anesthesia monitoring. Our patients either received propofol or sevoflurane as primary anesthetic agent and the age span of the patients was rather wide. Since both age [42, 43] and anesthetic agent [44] can influence the (frontal) EEG our results present a broad overview of the performance of the qCON. In order to evaluate the performance in a more detailed way, future studies of adequate sample size are necessary. Further, the electrode positions during EEG recording may have been slightly different from the qCON electrode locations. Published results describe that BIS values recorded from frontal and postauricular montages are similar [45]. Further, with our approach we could only investigate the qCON performance during the transitions without any neuromuscular blockade. Since relaxation can influence EEG-based monitoring as shown for the BIS [9, 10] we cannot draw the conclusion of how the qCON would react in a situation of possible intraoperative awareness during neuromuscular blockade.

In summary, we could show that the qCON seems to perform in a similar fashion when compared to other devices. We could highlight, as we have previously shown for other monitors, that the index (qCON) presents a considerable time delay during sudden state transitions, simulated by concatenation of EEG segments reflecting a stable qCON at the different levels *awake/sedation*, *adequate anesthesia*, and *suppression*. Further, the performance for capturing the state transitions is rather poor, most probably caused by the combination of time delay and the highly dynamic nature of the EEG during transitions in and out of responsiveness.
